# O.1.3-7 Hungarian Active Lifestyle (HEPA) Strategy 2033

**DOI:** 10.1093/eurpub/ckad133.104

**Published:** 2023-09-11

**Authors:** Gyöngyvér Lacza

**Affiliations:** Hungarian University of Physical Education, Hungary; gyongyver.lacza@gmail.com

## Abstract

Inactivity and obesity crisis is getting serious in Hungary in the last decade. According to the latest Eurobarometer studies (2022) 59% of the population are inactive and do not meet the WHO minimum physical activity recommendations. Besides, research shows that nearly 60% of Hungarian adults are overweight or obese, and every fifth child as well. As a respond to these challenges in 2023 the first ever Hungarian Active Leisure (HEPA) strategy has been prepared. As a first step we have created a sectoral map and interviewed potential action partners, including all the most recognized stakeholders from the recreation, sport, health and education sector. After a deep overview of the relevant literature, international guidelines and best practices the team of experts chose the OECD/WHO ‘Policy options to increase PA intervention’ model to follow (2023) as theoretical framework for future interventions. The major focus areas of interventions such as School-based interventions, Workplace-based interventions, Interventions of the Healthcare settings, Interventions in the sports sector and Information and communication policies must have been extended with three more areas: Urban design and transport policies, Natural environment and at last Seniors and Socially and economically disadvantaged citizens. A number of programs have already been piloted (just like Active School Program, Sport Clubs for Health, Workplace interventions or Senior - Pre-school children’s recreational-sport activities together) with great results. Indicators are not only the growing numbers of participants but also successful pilot programs are spreading and more and more work-places, local communities and sport clubs open up to follow these examples all over the country. Hopefully, in the medium and long term both physical activity rates will increase and health and well-being benefits are going to be significant too.

Sources: https://sport.ec.europa.eu/news/new-eurobarometer-on-sport-and-physical-activity

https://read.oecd-ilibrary.org/social-issues-migration-health/step-up-tackling-the-burden-of-insufficient-physical-activity-in-europe_540a3e56-en#page1

